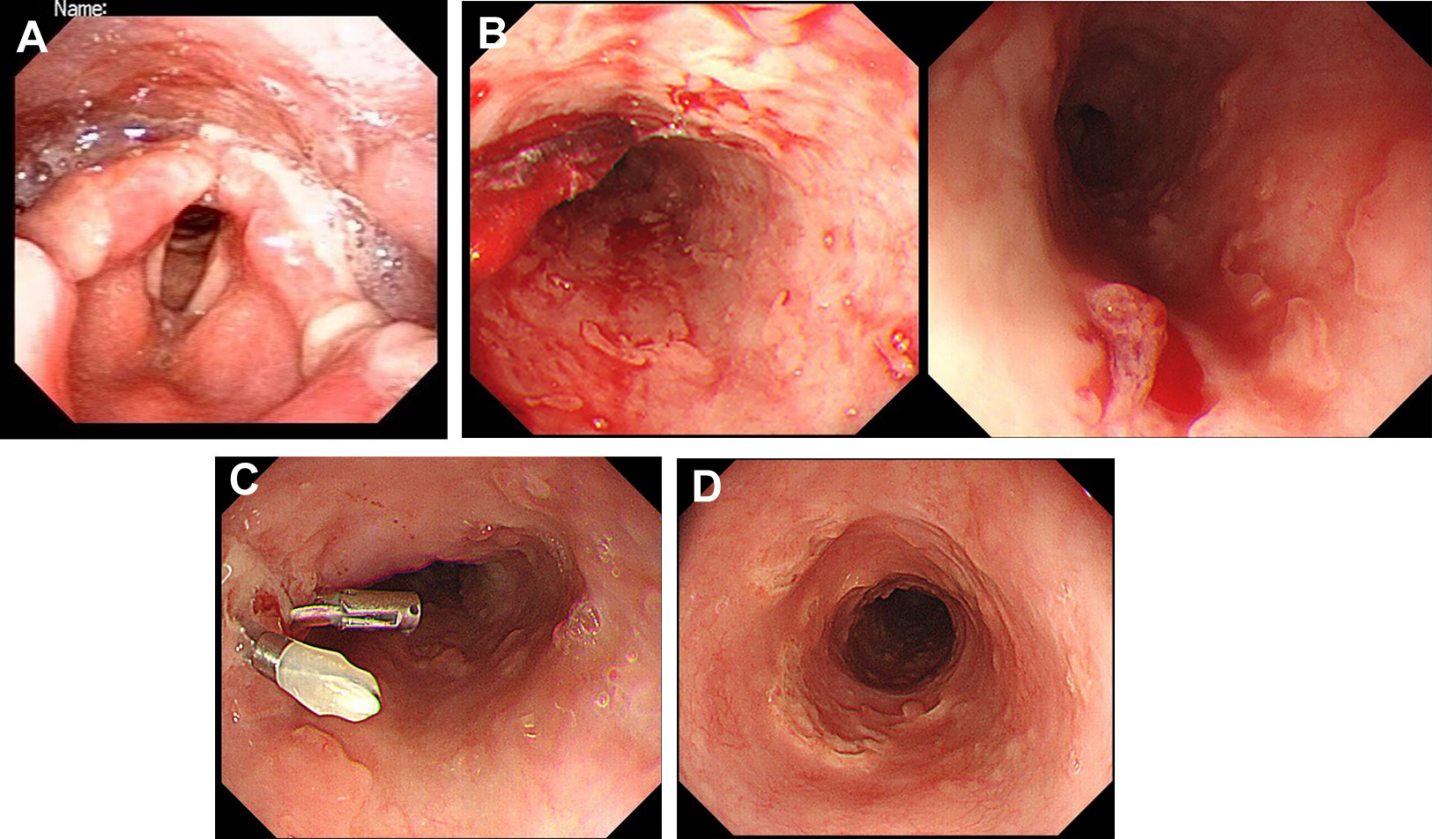# Herpes Simplex Esophageal Ulcer in an Immunocompetent Adult With Hematemesis

**DOI:** 10.1016/j.gastha.2022.09.012

**Published:** 2022-09-30

**Authors:** Yusuke Nomoto, Makoto Furihata, Taro Osada

**Affiliations:** Department of Gastroenterology, Juntendo University Urayasu Hospital, Chiba, Japan

A 61-year-old man with pharyngitis and stomatitis for 2 months was admitted due to difficulty in swallowing solids. Laryngoscopy demonstrated multiple laryngopharyngeal ulcers with exudative laryngopharyngitis (Figure A). He had elevated IgM antibody for herpes simplex virus (HSV). Acyclovir (750 mg daily) was orally administered soon after admission. After 2 days, he had hematemesis and tarry stool. Hemoglobin level decreased to 4.9 g/dL with hemorrhagic shock and required blood transfusion. Urgent upper gastrointestinal endoscopy showed multiple erosions and ulcers with spurting bleeding in the upper esophagus (Figure B). Multiple skipping erosions and round-shaped ulcers were spread 10 cm-wide. Temporal hematemesis is achieved by endoscopic clips to a visible vessel (Figure C). Punched-out ulcers remained but bleeding arrest was reconfirmed by follow-up endoscopy 3 days later. Sixty days post-discharge, endoscopy showed multiple scaring replaced the lesions (Figure D). His symptoms disappeared completely over time, with no recurrence so far. Our report gives a clinical description of exceedingly rare HSV esophageal ulcers accompanying hematemesis in an immunocompetent individual. Though the patient took warfarin and aspirin for old myocardial and middle cerebral infarction, he was not deemed immunocompromised. We propose esophagitis due to HSV should be considered a causative factor for hematemesis even in an immunocompetent host.

**Figure:**